# Permanganate of Potash

**Published:** 1884-12

**Authors:** 


PERMANGANATE OF POTASH.—Its Action and Uses, by Roberts Bartholow, M. D., L. L. D. From the *Medical News*

—a Reprint. This small volume furnishes an exhaustive treatise on the effects of this valuable remedy and its employment in various affections.

